# Hostility and Cognitive Complexity: A Meta-analysis

**DOI:** 10.11621/pir.2025.0108

**Published:** 2025-03-01

**Authors:** Gleb D. Emelin, Sergey N. Enikolopov

**Affiliations:** a Lomonosov Moscow State University, Russia; b Russian Presidential Academy of National Economy and Public Administration, Moscow, Russia; c Mental Health Research Center, Moscow, Russia

**Keywords:** hostility, cognitive complexity, integrative complexity, cognitive simplicity, meta-analysis

## Abstract

**Background:**

We can see outbreaks of social violence (notably wars, riots, and revolutions), both historically and in the current social situation. Some authors point to the impact of hostility on human cognitive processes and on decision making, and through these factors — on aggressive behaviour. Moreover, some retrospective studies note the role of cognitive complexity in the peaceful resolution of conflicts (including international conflicts). These findings prompted us to ask whether the two phenomena are related. To answer this question a meta-analysis of correlation between hostility and cognitive complexity was conducted.

**Objective:**

Our objective was to summarise research findings on the relationship between hostility and cognitive complexity.

**Design:**

The total number of papers screened was 839 (in English and Russian). A total of 5 effect sizes from 4 selected papers were included in the meta-analysis derived from a pooled sample of 3114 participants. Three-level random-effects meta-analysis, p-curve, p-uniform and p-uniform* methods were used in the data analysis.

**Results:**

The results of different effect size calculation methods (three-level random-effects model, two-level random-effects model, and p-uniform*) confirmed that there is a moderately weak negative correlation between hostility and cognitive complexity. The most accurate result is r_pooled_ = –.22 [–.45; .003].

**Conclusion:**

There is a negative correlational relationship between hostility and cognitive complexity. Apparently, hostility and cognitive complexity have some reciprocal influence on each other. The authors hypothesise that hostility is rather complementary to cognitive simplicity as the opposite pole of cognitive complexity - if the function of hostility in ensuring the protection of one’s sense of Self is taken into account. Both hostility and cognitive simplicity work to create a simple (in one case dangerous, in another case unambiguous) world in which it is easier to make decisions (including the decision to engage in aggressive behaviour).

## Introduction

Hostility is proposed to be one of the key factors that ensures the implementation of aggressive behaviour on an individual level (Smeijers, 2023). Consequently, hostility seems to have an impact on the increase of social violence observed both in the historical perspective and in the current social situation. This influence can be analysed through the framework of considering hostility as a cognitive phenomenon that affects information processing and subsequent decision making.

According to the literature analysis, in most cases the term “hostility” refers to the nature of a certain phenomenon rather than an independent psychological reality (*i.e.*, a system of attitudes, cognitive distortions etc.). In other words, hostility acts as a functional “modifier” of cognition. According to Crick and Dodge (1994), the main function of this “modification” (as well as of the phenomenon of human aggression in general) is to protect one’s sense of Self. A possible explanation of how hostility contributes to the defense of one’s Self is by providing subjects with clear information in an uncertain and ambiguous social environment (Smeijers et al., 2019).

Aggression, hostility and anger are often presented as interrelated phenomena. This logic of consideration was suggested in the 1960s by the prominent American psychologist Arnold Buss (Buss, 1961). He believed that aggression as a phenomenon consists of three components: anger as an emotional component of aggression, physical and/or verbal aggression as a behavioural component, and hostility as a cognitive component. It is possible to say that there is some “consensus” regarding the “cognitive nature” of hostility. A large number of studies with different operationalisations of hostility have been conducted *i.e.* hostility as a personality trait (*e.g.*, Anderson et al., 1996), a system of attitudes (*e.g.*, Smith, 1992, Barefoot, 1992), an “image of the world” (*e.g.*, Enikolopov & Chudova, 2017), and a cognitive distortion (*e.g.*, Dodge et al., 1990). It is important to say that the operationalisation of hostility as a cognitive distortion is now the most popular and this approach has made some very significant contributions to the issue of understanding the essence of hostility.

The origins of the hostile cognitive distortions approach or the hostile biases approach was established within Kenneth Dodge`s framework. Dodge and Crick proposed a nonlinear cyclical model of social information processing (SIP) (Crick & Dodge, 1994). According to this model, children learn how to use aggressive behavior due to the hostile distortion at one of the social information processing stages. The model posits that human beings go through a series of stages when faced with a social cue. First, they encode the situation (Stage 1), taking in relevant information. Next, they create a *mental representation* of the situation (Stage 2), interpreting the cues and relating them to past experiences. The child then accesses or constructs potential *responses* from their repertoire (Stage 3). A *response decision* follows (Stage 4), where the child evaluates the potential consequences of each response and chooses one. Finally, the chosen response is *enacted* (Stage 5). Dodge`s original model presents these steps as a sequence.

The revised model retains these core processing steps but emphasises the cyclical and recursive nature of social information processing during the social interaction. It acknowledges that a child’s initial response elicits a reaction from others, which then becomes new social information to be processed by the child, restarting the cycle. Also the child’s internal database has a very important role in this model — specifically their store of memories, social knowledge, and social schemas — all of which influences each step of the processing sequence. The model also takes into account the importance of clarifying goals within the social situation (*i.e.*, “how my peers will assess my action?), as these goals shape how the child interprets the cues and selects a response. For example, aggressive children might misinterpret ambiguous cues as hostile, struggle to generate non-aggressive responses, or overestimate the positive consequences of aggression.

It is noted that the hostile schema which occurs in childhood plays an important role in the development of a hostile encoding pattern and consequently, hostile biases (Crick & Dodge, 1994, Smeijers Bulten & Brazil , 2019). The schema itself is a complex mental phenomenon that emerges from memories, emotions, cognitions, self-attitudes and attitudes toward others (Smeijers et al., 2019). The hostile schema is a specific pattern of information perception. According to the schema-inconsistent hypothesis, the hostile schemas direct one`s attention not on the expected hostile social cues, but rather on the schema-inconsistent information (*i.e.* non-hostile information) because this information resonates with expectations.

A fundamental problem in hostility research is that the phenomenon itself has not been sufficiently reflected on from a theoretical and methodological point of view. A broad diversity of “hostile” entities distinctly demonstrates this problem. Moreover, there is a lack of evidence on how the different ways to operationalise hostility relate. There is some empirical evidence from eye-tracker studies that show aggressive people linger meaningfully longer on non-hostile information than non-aggressive people. In turn, non-aggressive people meaningfully hold their gaze longer on hostile information than aggressive people. In other words, people with a stable hostile schema require more cognitive effort to incorporate non-hostile information into the perception of social information (Horsley, de Castro & der Schoot, 2010).

This perspective allows us to fit hostility into the space of human cognitive life and raise simple analytical questions. In particular, within this paper, we will explore the relationship between hostility and cognitive complexity.

### Hostility and cognitive complexity

Peter Suedfeld and Philip Tetlock were probably the first researchers who addressed the issue of the relationship between hostility and cognitive complexity. Their research is located at the intersection of conflictology, political science and psychology. One of the central problems of this research line is the problem of the role of “integrative complexity” in international conflict processes (Suedfeld, Tetlock & Ramirez, 1977). The concept of integrative complexity is a development of the concept of cognitive complexity-simplicity (Biery, 1955, Scott, 1962). The main difference between the concept of integrative complexity and the original concept^1^ of cognitive complexity is that in addition to the differentiation of information, perspectives, or dimensions that are processed in relation to a concrete problem (differentiation), integrative complexity also includes the perception and understanding of connections between divergent dimensions (integration) (Békés & Suedfeld, 2020). Interesting results have been obtained about the relationship between integrative complexity and the situation of armed conflict. In the study of the political rhetoric of the Soviet Union and the US (1945–1983), it was found that the integrative complexity of foreign policy statements from officials decreased significantly in the situation of the outbreak of war (Tetlock, 1985, 1988). In particular, integrative complexity might be a significant predictor of military or peaceful outcomes of international crises (Conway III, Suedfeld & Tetlock, 2001).

Research on the need for cognitive closure is also an area on the intersection of hostility and cognitive complexity. The need for cognitive closure appears when there is an increased need for simple answers that can reduce uncertainty (Kruglanski & Fishman, 2009). There is evidence that in post-war periods, the need for cognitive closure often escalates authoritarian and extremist attitudes (Nestik, 2023). In other words, cognitively simple answers to cognitively simple questions lead to increased political and national hostility.

Before describing the methods, we will define hostility and cognitive complexity. Hostility is understood as a stable complex system of worldviews, which manifests itself in the overwhelming evaluation of the external world and surrounding people as posing a danger to the subject. Due to the stability of this system, it can be operationalised in the study as trait hostility (Enikolopov & Chudova, 2017).

I n turn, we define cognitive complexity through the conception of integrative complexity as a stable pattern of information processing that includes two aspects: differentiation and integration. Differentiation refers to the breadth of perceptions of different dimensions and perspectives when considering a problem. Integration refers to the ability to create a holistic image from these dimensions and perspectives (Békés & Suedfeld, 2020).

The idea for this paper was born from the suggestion that hostility should be negatively correlated with cognitive complexity because hostility (and related cognitive biases) should construct a simple black-and-white reality. The main research question was formulated as follows: “Is there a relationship between hostility and cognitive complexity?”

We also thought about possible mediators of the relationship between hostility and cognitive complexity, although it was not possible to implement a mediator analysis in this study. The role of gender in the manifestation of hostility is a debatable issue. Early studies noted that there are no differences in hostility between men and women (Enikolopov & Tsibulsky, 2007, Moreno et al., 1994). However, modern systematic reviews do not give such an unambiguous answer. In a number of studies, differences are still found, and both in the direction of greater hostility of men and women.

In some studies, it is noted that hostility begins to “control” social-perceptual processes more strongly during the course of life experience and adulthood. In the same study, adults found a link between hostility and failures in information processing and perception of the world as uncertain and complex, while no such link was observed in young people (Enikolopov & Chudova, 2017).

In the context of social information processing concepts (N. Crick, K. Dodge, D. Smeijers, etc.), these findings are quite logical, since the source of hostility is a “database” that includes accumulated experience, gender socialisation, etc.

To our surprise, we found no studies of the correlation between hostility and cognitive complexity in our initial search. Because of that the decision was made to use meta-analysis to locate studies and determine the generalised strength of the relationship between these two variables.

## Methods

### Study Selection

The aim of the study was to include empirical research of the relationship between cognitive complexity and hostility conducted from 1950^2^ to 2024.

Overall, three databases were used to search for papers: Google Scholar, ScienceDirect, and PubMed. The search was iterative, using the keywords denoting hostility (“trait hostility”, “hostil*”, “hostility”, “hostile”, and “враждебность”) and cognitive complexity (“cognitive complexity”, “cognitive complexity-simplicity”, “cognitive simplicity”, “cognitive simplicity-complexity”, “integrative complexity”, “conceptual complexity”, "κогнитивная сποжность", "κогнитивная простота-сπожность", "κогнитивная сποжность-простота", "κогнитивная простота", "концептуаπьная сποжность", and "интегративная сποжность"). Keywords and databases were selected by authors. The total number of papers screened was 839. The criteria for initial screening of papers were as follows: relevance to the research questions, non-clinical status of participants, method of measuring effect size (correlation), publication in English or Russian languages, and absence of a paywall.

**Figure 1:**
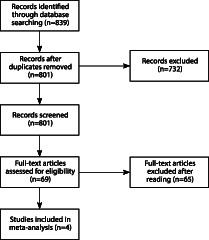
Flowchart of the meta-analysis study selection process

*Figure 1* shows the study selection process (Mohrer et al., 2009).

The following items were prescribed for the included studies: authors, year of publication, way of operationalisation of hostility, hostility measure, way of operationalisation of cognitive complexity, cognitive complexity measure, raw effect size (correlation coefficient), corrected effect size, mean age, of the sample, gender of participants.

Four papers including a total of five effect sizes were selected for a detailed examination of subsequent inclusion in the meta-analysis.

**Table 1:** Characteristics of studies included in meta-analysis

Authors	Hostility (Measure)	Complexity Cognitive (Measure)	Raw effect size	N	Mean age	Sex
Bruch, McCann & Harvey, 1999	Type A Behaviour (JAS)	Cognitive Differentiation (Listing and Comparing of Attributes)	–.28	67	19	Males only
Bruch, McCann & Harvey, 1999	Type A Behaviour (JAS)	Cognitive Integration (Paragraph Completion Method)	–.49	67	19	Males only
Malesza & Kaczmarek, 2018	Vulnerable Narc. (HSNS)	Cognitive Complexity (reversed scale) (BIS-11)	–.24	337	23.1	Both
Sillars & Parry, 1982	Other-directed attributions of blame (16 roommate grievances questionnaire)	Communicative Complexity (Paragraph Completion Method)	–.17 (ns)	78	19	Both
Kapitány-Fövény et al., 2020	Hostility (BSI)	Cognitive Impulsivity (BIS-11_Hungarian)	–.06 (ns)	2632	40.3	Both

*Note. ns = non-significant*

### Statistical Analyses

#### Calculating Effect Sizes

All raw effect sizes were Z-transformed (Rosenthal, 1991).

#### Meta-analytic integration

A three-level random-effects model (Harrer et al., 2021) was used to elucidate an association between hostility and cognitive complexity. This model was chosen because two of the five available effect sizes were extracted from the one paper. The classic meta-analytic random-effects model builds on the logic of the existence of two error levels of the true effect size. The first level of error, or “participant level”, reflects a sampling error or a deviation from the true effect size due to the data being collected in a single study. The second level of error, or “study level”, reflects heterogeneity between the studies included in the meta-analysis.

For a classic meta-analysis, an important assumption is that studies are fundamentally independent from each other. If several effect sizes were extracted from a single study, this could distort the real level of heterogeneity of studies. However, this problem is solved in three-level models. The third level of error, or “cluster level” (which can be either individual studies or subsets of studies), reflects heterogeneity between clusters.

Thus, in our study we used three-level random-effects model due to: 1) the expected heterogeneity associated with different operationalisation of the cognitive complexity and hostility; 2) two effect sizes were extracted from the same study. The method we used to estimate the between-study heterogeneity is multilevel version of I^2^ (Cheung, 2014).

#### Analysis of moderators

Sex, age and way of operationalisation of hostility and cognitive complexity were chosen initially as moderators, but due to the limited number of included papers, it was decided to drop the moderator analysis due to inability to conduct a moderation analysis because of the small number of effect sizes.

#### Publication bias analysis

The p-curve (Simonsohn, Nelson & Simmons, 2014) and p-uniform (van Aert & van Assen, 2018; van Aert, Wicherts & van Assen, 2016) methods were used to test for publication bias. The p-uniform* is a more accurate modification of p-uniform because it incorporates insignificant effect sizes in the calculations. In addition, p-uniform* does not overestimate the effect size.

#### Software for data analysis

The R programming language and RStudio environment with metafor, meta, dmetar, and puniform packages were used for statistical data processing.

## Results

A total of 5 effect sizes were included in the meta-analysis derived from a pooled sample of 3114 participants, and 84.5 was a sample of one of the four papers.

### Three-level meta-analysis

Due to the severe external heterogeneity of the data (almost the whole study is unique in the methods used and operationalisation of the constructs), the usage of multivariate meta-analytic models seemed to be adequate.

**Table 2:** Distributions of studies by clusters, effect sizes and their variance

Number of cluster	Study	Effect size (Zrs)	Variance of Zrs
1	Bruch, McCann & Harvey, 1999	–.29	.015
1	Bruch, McCann & Harvey, 1999	–.54	.014
2	Malesza & Kaczmarek, 2018	–.24	.002
3	Sillars & Parry, 1982	–.17	.013
4	Kapitány-Fövény et al., 2020	–.06	.0004

As can be seen in the table, the first and second effect sizes were distributed in one cluster.

**Table 3:** Results of evaluation of the components of the variance of the true effect

τ^2^	Est.	Number of groups on level
τ^2^_level 3_	.0133	4
τ^2^_level 2_	.0088	5

**Figure 2:**
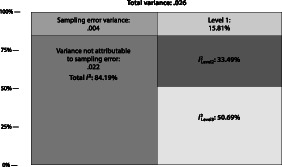
Distribution of the total heterogeneity variance

*Figure 2* shows the distribution of explained variance of heterogeneity across levels (Cheung, 2014).

As can be seen from the figure, half of the heterogeneity is explained by differences between studies and a third of the variance is explained by features within studies.

*Table 4* presents the results of the three-level random-effects meta-analysis and the results of the two-level classical random-effects meta-analytic model. *Table 5* presents the results of statistical comparison of these models with each other.

**Table 4:** Three-level random-effects meta-analysis and two-level random-effects meta-analysis results (k=5)

Type of model	Pooled Zr (pooled r)	ES (k)	Samples	95 CI
Three-level random-effects model	–.22 (–.22)	5	4	[–.45; .003]
Two-level random-effects model	–.24 (–.23)	5	5	[–.45; –.02]

**Table 5:** Comparison of three- and two-level meta-analysis models

Type of model	df	AIC	BIC	AICc	logLik	χ^2^_1_	p-value
Three-level random-effects model	3	3.24	1.4	27.24	1.4		
Two-level random-effects model	2	1.5	0.24	13.5	1.3	.23	.633

As can be seen from the comparative analysis data, no statistically significant differences were found between the models.

### Publication bias data analysis

Methods based on p-values were used to test for publication bias.

**Table 6:** P-curve analysis results

Type of test	p_binomial_	Full Curve		Half Curve		Evidential Value		True effect size (Cohen`s d)
		Z_full_	P_full_	Z_half_	P_half_	present	inadequate	
Right-Skewness test	.062	–4.454	< .001	–3.471	< .001	Yes	No	
Flatness test	1	2.733	.997	3.682	> .999	Yes	No	.402

The p-curve analysis results indicate that the data are generally undistorted. This conclusion is supported by the p-uniform and p-uniform* methods (*Table 7*).

**Table 7:** P-uniform and p-uniform* methods results

Type of test	Test of no effect	Publication bias test
p-uniform	z = –2.65, p = .004	z = –.836, p = .8
p-uniform*	z = .8, p = .09	–

As can be seen from the table, the two related methods gave slightly different results. In the first case, it can be said that the null hypothesis of no effect (*i.e.*, distortion of any nature) is rejected, but in the second case the situation is the opposite. In this case it makes sense to rely on p-uniform* data as a more accurate method of distortion estimation. Overall, publication bias also was not detected.

Thus, we can conclude that our data are ***statistically*** free from distortions.

### Overall effect size

Due to the strong heterogeneity of the studies, it was decided to combine the outcomes of the different effect size estimation methods.

**Table 8:** Overall effect size according to four different calculation methods

Method of effect size calculation	Effect Size (r) and CI
Three-level random-effects model	–.22 [–.45; .003]
Two-level random-effects model	–.23 [–.45; –.02]
P-uniform* (estimator: ML)	–.143 [–.37; .39]
P-uniform* (estimator: P)	–.196 [–.46; .151]

Overall, it can be said that there is a moderately weak negative correlation between hostility and cognitive complexity.

## Discussion

The results of the literature search for inclusion in this meta-analysis characterise not only a particular area of research on the relationship between hostility and cognitive complexity, but also the problem of research on hostility overall. Hostility as an object of psychological research is less popular than anger or aggression, so the difficulties we faced during literature search are legitimate. It can be honestly said that this meta-analysis is heavily cluttered with different ways of operationalising hostility. For example, hostility was operationalised as an independent psychological phenomenon only in one study^3^. In most studies, however, hostility appears as an important, but far from independent, part of larger constructs such as A-type behaviour, vulnerable narcissism, or the attitude of blaming others for everyday problems. The situation with cognitive complexity is slightly better, but there is also a strong confusion of concepts from integrative to communicative complexity, and ways of operationalisation as some ability that can be assessed externally to self-reported cognitive complexity. Of course, this situation makes it difficult to draw conclusions, although modern statistical methods do produce relatively accurate results despite the strong heterogeneity between studies.

It was not possible to conduct mediator analysis due to the fact that each study was categorised into groups according to their moderator. The multivariate metaanalysis method allows the avoidance of biases caused by dependent outcomes, which in the case of 2 out of 5 effect sizes poses high risks. Although comparative analysis did not show a significant difference between the three-level and two-level models, the 3-level model cannot be rejected due to our empirical knowledge of the relatedness of 40 of the effect sizes.

The p-values analysis showed that our data, at least statistically, did not fall victim to publication bias. These results are convergently confirmed by the two methods. It also confirms our thesis about the unpopularity of the topic of hostility.

Due to the high heterogeneity, we decided to use multiple methods of effect size calculation at once. The results showed that the relationship between hostility and cognitive complexity ranged from –.143 to –.23, indicating a moderately weak negative relationship between the variables.

If we turn to the analysis of confidence intervals (*Table 8*), it can be seen that the effect size obtained from the p-uniform* method using different estimators gives a broad range. Therefore, the results of three-level random-effects meta-analysis seem to be more accurate. Thus, it can be said that the correlation between hostility and cognitive complexity is closer to moderately negative.

It is possible that hostility and cognitive complexity have some reciprocal influence on each other: the lower the cognitive complexity, the greater the hostility, and vice versa. Without speculating in this paper about causal relationships between the variables, let us clarify the thesis which has been stated at the beginning: Hostility is rather complementary to cognitive simplicity as the opposite pole of cognitive complexity, if the function of hostility in ensuring the protection of one’s sense of Self is taken into account.

Given that hostility in a certain way constructs a dangerous world image (Enikolopov & Chudova, 2017; Wang & Xia, 2019), this world is simultaneously more comprehensible in the sense that there are one’s own and strangers, and it is necessary to always be prepared and to defend oneself. In its turn, cognitive simplicity provides a limited number of categories for assessing the external environment and simplified patterns of information integration due to which a black-and-white world is established. And this black-and-white image of the world is very consonant and complementary to the hostile dangerous image of the world.

Currently, many questions remain about the nature and presence of this relationship in children, for example the presence or absence of sex differences, the nurture or nature question, and the distinction of cause and effect in this correlation. However, the result obtained appears to be the first attempt to generalise the small amount of scientific knowledge on this topic.

## Conclusion

Given the “cognitive” way to the research on hostility is now prevalent in science, the results of our meta-analysis are important in context of future theoretical and empirical developments in the psychology of hostility. Our goals were to summarise the existing literature on the topic of the relationship between hostility and cognitive complexity, and to clarify whether there is a relationship between these two variables. Our results led us to a conclusion that there is a moderately negative correlational relationship between hostility and cognitive complexity (r = –.22). The small number of published studies gives freedom for creative search. Therefore it is necessary to continue research in this area to understand the place and role of hostility in the space of human cognition.

## Limitations

The main limitation of the study can be considered to be the lack of access to the full texts of the dissertations because there may be data of interest in the context of this problem.

Another limitation is the choice of only two publication languages, as it is possible that there may be published evidence in languages other than Russian or English.

## Footnotes

1 There are many approaches that are quite synonymous with each other within the study of complexity as a psychological phenomenon: cognitive complexity, integrative complexity, conceptual complexity, etc. (Modrick, 1992).

2 This limit was set according to the bibliographical data: Ermakov, P. N., Abakumova, I. V., Fedotova, O., & Shchetinina, D. P. (2016). Hostility as a Psychological Phenomenon and Object of Scientific Research. *International Journal of Environmental and Science Education*, *11*(18), 10829–10837.

3 To be accurate, hostility in this research (Kapitány-Fövény et al., 2020) was operationalised not as a personality trait, but rather as a particular psychological symptom.
